# Lipid‐Peptide‐mRNA Nanoparticles Augment Radioiodine Uptake in Anaplastic Thyroid Cancer

**DOI:** 10.1002/advs.202204334

**Published:** 2022-12-01

**Authors:** Qinglin Li, Lizhuo Zhang, Jiayan Lang, Zhuo Tan, Qingqing Feng, Fei Zhu, Guangna Liu, Zhangguo Ying, Xuefei Yu, He Feng, Heqing Yi, Qingliang Wen, Tiefeng Jin, Keman Cheng, Xiao Zhao, Minghua Ge

**Affiliations:** ^1^ Department of Head and Neck Surgery Center of Otolaryngology-head and neck surgery Zhejiang Provincial People's Hospital (People's Hospital of Hangzhou Medical College) Key Laboratory of Endocrine Gland Diseases of Zhejiang Province Hangzhou Zhejiang 310014 China; ^2^ The Cancer Hospital of the University of Chinese Academy of Sciences (Zhejiang Cancer Hospital) Institute of Basic Medicine and Cancer (IBMC) Chinese Academy of Sciences Hangzhou Zhejiang 310022 China; ^3^ CAS Key Laboratory for Biomedical Effects of Nanomaterials & Nanosafety CAS Center for Excellence in Nanoscience National Center for Nanoscience and Technology Beijing 100190 China; ^4^ Center of Materials Science and Optoelectronics Engineering University of Chinese Academy of Sciences Beijing 100049 China

**Keywords:** anaplastic thyroid carcinoma, lipid‐peptide‐mRNA nanoparticles, mRNA delivery, sodium iodide transporter

## Abstract

Restoring sodium iodide symporter (NIS) expression and function remains a major challenge for radioiodine therapy in anaplastic thyroid cancer (ATC). For more efficient delivery of messenger RNA (mRNA) to manipulate protein expression, a lipid‐peptide‐mRNA (LPm) nanoparticle (NP) is developed. The LPm NP is prepared by using amphiphilic peptides to assemble a peptide core and which is then coated with cationic lipids. An amphiphilic chimeric peptide, consisting of nine arginine and hydrophobic segments (6 histidine, C18 or cholesterol), is synthesized for adsorption of mRNA encoding NIS in RNase‐free conditions. In vitro studies show that LP(R9H6) m NP is most efficient at delivering mRNA and can increase NIS expression in ATC cells by more than 10‐fold. After intratumoral injection of NIS mRNA formulated in optimized LPm NP, NIS expression in subcutaneous ATC tumor tissue increases significantly in nude mice, resulting in more iodine 131 (^131^I) accumulation in the tumor, thereby significantly inhibiting tumor growth. Overall, this work designs three arginine‐rich peptide nanoparticles, contributing to the choice of liposome cores for gene delivery. LPm NP can serve as a promising adjunctive therapy for patients with ATC by restoring iodine affinity and enhancing the therapeutic efficacy of radioactive iodine.

## Graphical abstract

Lipid‐peptide‐mRNA (LPm) nanoparticles (NPs), is employed to deliver mRNA. mRNA encoding sodium iodide symporter (NIS) delivered by LPm NPs significantly augment the expression of NIS protein on the cell membrane of anaplastic thyroid cancer (ATC) in vivo and in vitro. NIS‐mRNA LPm NPs combining 131I therapy heightens intracellular aggregation of 131I and antitumor effect.

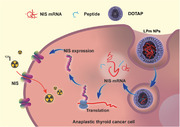

## 1 Introduction

Thyroid cancer has been continuously increasing in worldwide incidence for the past few decades, accounting for more than 90% of the malignant tumors of the endocrine system.^[^ ^1^, ^2^, ^3^ ^]^ Notably, anaplastic thyroid cancer (ATC) is the most aggressive and lethal form of thyroid cancer, with a mortality rate approaching 100% and a median survival time of 4–6 months.^[^ ^1^, ^4^ ^]^ Different from differentiated thyroid cancer (DTC) with good prognosis, ATC patients usually show rapid growth and invasion of neck masses, and involvement of local cervical lymph nodes. ATC's rapid invasion of adjacent tissues of the neck and cervical lymph nodes will lead to local compression symptoms, such as dysphagia, dyspnea, and neck pain, which are usually difficult to resolve by surgery.^[^ ^5^, ^6^, ^7^ ^]^ Current treatments for thyroid cancer rely on various combinations of surgical resection with adjuvant therapies, such as radioiodine (^131^I), but have shown only limited survival benefits on patients with ATC.^[^ ^8^, ^9^, ^10^ ^]^ The loss of iodide uptake capacity in ATC is mainly due to the reduced/lost functional expression of the sodium iodide symporter (NIS), which is an important therapeutic target for the treatment of ATC.

NIS is a glycoprotein expressed on the basement membrane of thyroid cells, which mediates the active transport of iodide into thyrocytes for the biosynthesis of thyroid hormones.^[^ ^8^, ^11^ ^]^ NIS is critical for the diagnosis and therapeutic management of thyroid diseases, including thyroid carcinoma. Induced gene expression and gene delivery are two common methods used to increase NIS expression. The expression of NIS protein induced by some small molecule drugs, such as capsaicin and tunicamycin, has been confirmed, but this method lacks specificity of induction and is not applicable for different cancers.^[^ ^12^, ^13^, ^14^, ^15^, ^16^ ^]^ Through targeted transfer and expression of the NIS gene, radioiodine treatment was used to treat a variety of tumors. The protein expression efficiency by gene therapy is mainly influenced by the design and selection of the delivery vehicles.^[^ ^9^, ^17^, ^18^, ^19^ ^]^ In addition, DNA cannot be transcribed into Messenger RNA (mRNA) until it enters the nucleus, and the method of DNA transfection may increase the risk of genomic integration and mutagenesis.^[^ ^20^ ^]^ Recently, chemically modified mRNA has received extensive attention in tumor therapy as an alternative to protein‐based drugs. Since the chemically modified mRNA can be translated into the target protein as long as it is internalized into the cytoplasm, the mRNA transfection efficiency is high and the potential risk of foreign gene integration into the genome is avoided.^[^ ^21^, ^22^ ^]^ In addition, mRNA can be produced in a cell‐free environment by in vitro transcription (IVT), thereby avoiding the quality and safety issues associated with production using microorganisms or cultured cells.^[^ ^23^ ^]^ Compared with proteins and viruses, the mRNA production process is simpler and less expensive, and will be easier to achieve industrial‐level production. The premise of mRNA functioning is that it needs to enter the cell. In the absence of a delivery system, the mRNA itself is difficult to carry across the cell membrane barrier and is easily degraded.^[^ ^23^ ^]^ Therefore, the design of safe and efficient delivery vehicles has become the key to the success of mRNA therapy.

The ability of nanomaterials to safely and effectively protect the loaded nucleic acids, especially mRNA, has been confirmed in basic and clinical studies.^[^ ^23^ ^]^ Currently, a variety of nanocarriers have been developed for in vivo delivery of mRNA.^[^ ^25^, ^26^, ^27^ ^]^ Among them, lipid‐based gene delivery vehicles are the most widely studied. Cationic liposomes have high nucleic acid adsorption efficiency and high transfection efficiency in vitro, but the adsorbed nucleic acids are exposed on the outside and cannot be used for in vivo delivery of nucleic acids. Lipid nanoparticles (LNP) have been developed with mature technology and are in clinical trials,^[^ ^28^ ^]^ but the synthesis of LNP requires advanced equipment and the development cost is also high. Lipid/calcium/phosphate (LCP) NPs and liposome‐protamine‐DNA (LPD) NPs have been designed for in vivo targeted delivery of nucleic acids to the tumor. However, the metabolism of calcium phosphate in vivo may cause additional burden for patients and the immunogenicity of protamine may result in rapid clearance of nanoparticles.^[^ ^29^ ^]^ Thus, there is an unmet need to develop a new core for nucleic acid transport.

Herein, an optimized peptide core‐based cationic liposome, lipid‐peptide‐mRNA (LPm) NPs, was employed to deliver mRNA encoding NIS to ATC for improving the sensitivity of ATC to radioiodine treatment. We developed a series of arginine‐rich amphiphilic peptides as cationic liposome cores for adsorbing nucleic acids and confirmed their feasibility in gene delivery. During in vitro and in vivo experiments, the expression of functional NIS on ATC membrane was significantly increased, resulting in higher intracellular aggregation of ^131^I, achieving a good antitumor effect. LPm NPs combined with ^131^I may be a promising adjuvant therapy for ATC patients.

## 2 Results and Discussion

### 2.1 Low NIS Expression Level is Associated with Poor Prognosis in Patients with TC

**Figure 1:**
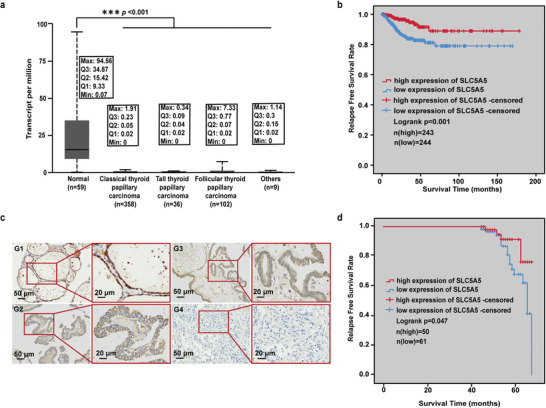
The relationship between the prognosis of TC patients and NIS expression. a) Expression of SLC5A5 in TC based on tumor histology (from UALCAN). Min, minimum; Q1, lower quartile; Q2, median; Q3, upper quartile; Max, maximum. b) The effect of SLC5A5 expression on RFS in TC patients (from cBioPortal database). Statistical significance was tested by two‐tailed Student's *t*‐test. c) Representative images of NIS protein immunohistochemical staining in thyroid tissues of different histological types. G1, NIS is expressed in the cell membrane of normal thyroid tissue; G2, NIS is expressed in the cell membrane of PTC sensitive to radioactive iodine therapy; G3, NIS protein is expressed at low level in the cell membrane of PTC resistant to radioactive iodine therapy; G4, NIS is not expressed in ATC (resistant to radioiodine therapy) (scale bar: 50 and 20 µm). d) Association of NIS expression levels with RFS in TC patients. Patients with NIS (*n* = 111) were stratified into two groups according to NIS IHC staining intensity. Patients with low NIS expression (intensity grade − and +) had worse recurrence‐free survival when compared with patients with high NIS (intensity grade ++ and +++). Statistical significance of Kaplan–Meier plots (b and d) was analyzed by log‐rank test.

NIS is a key protein for iodine uptake in thyroid cells. To understand the expression of NIS in TC patients and its relationship with prognosis, we used the University of Alabama at Birmingham CANcer (UALCAN) data analysis portal database to analyze the expression of NIS‐encoding gene SLC5A5 in thyroid cancer patients. Compared with normal thyroid tissue, the transcription level of SLC5A5 in thyroid cancer tissue was significantly decreased (*p* < 0.001) (Figure S1a, Supporting Information), and the transcription level of SLC5A5 in different types of thyroid cancer was decreased (*p* < 0.001) (**Figure** **1**a). Following the increase in individual cancer stages, the SLC5A5 transcript level also decreased (Figure S1b, Supporting Information). More importantly, Kaplan–Meier analysis based on the cBioPortal database showed that TC patients with high SLC5A5 transcript levels had significantly longer recurrence‐free survival (RFS) than patients with low SLC5A5 transcript levels (*p* = 0.001) (Figure 1b).

To further verify the expression level of NIS in TC patients and its relationship with prognosis, we examined the expression of NIS in 111 human thyroid specimens using immunohistochemistry. The results showed that the expression of NIS in the cell membrane decreased with increasing malignancy of the thyroid tumor. The NIS expression was low or absent in the iodine therapy‐resistant thyroid cancer, and NIS protein expression could not be found in the ATC tissue (Figure 1c). Thyroid cancer patients who were first treated in Zhejiang Cancer Hospital and underwent surgery from January 2016 to December 2017 were then followed up. Kaplan–Meier analysis showed that TC patients with high NIS expression had longer RFS than those with low NIS expression (*p* < 0.05) (Figure 1d). Taken together, our results suggest that lower expression of NIS in primary thyroid cancer is strongly associated with more aggressive features of the primary tumor, as well as worse prognosis and insensitivity to iodine therapy.

The normal expression of NIS on thyroid cell membrane can effectively mediate the accumulation of iodine activity into cells.^[^ ^30^ ^]^ The application of radioisotopes has made great progress in the diagnosis and treatment of thyroid cancer, but its effectiveness ultimately depends on the expression of functional NIS on the tumor cytoplasmic membrane, because the insufficient accumulation of radioactive iodine caused by the decline of NIS expression is the main reason for the failure of treatment.^[^ ^31^ ^]^ NIS gene expression is often down regulated in thyroid cancer. Among DTC with good prognosis, about 10–20% of patients are dedifferentiated due to epigenetic changes and signal pathway imbalance, which leads to increased malignancy and affects the expression of functional NIS, thereby reducing the efficacy of late DTC ^131^I treatment.^[^ ^32^, ^33^ ^]^ The expression of NIS is almost completely silent in poorly differentiated and anaplastic thyroid carcinoma with high malignancy.^[^ ^10^, ^34^ ^]^ This is also the same as our research results. The higher the degree of malignancy, the lower the expression of NIS in TC, the worse the prognosis of patients and the insensitivity to ^131^I treatment. Among them, there was no NIS expression in ATC which is the highest degree of malignancy, and ^131^I treatment was ineffective. Therefore, restoring NIS expression on ATC cell membranes to return the sensitivity of ATC patients to ^131^I treatment may be a practical and effective approach.

### 2.2 Rational Design of mRNA Peptides Core

**Figure 2:**
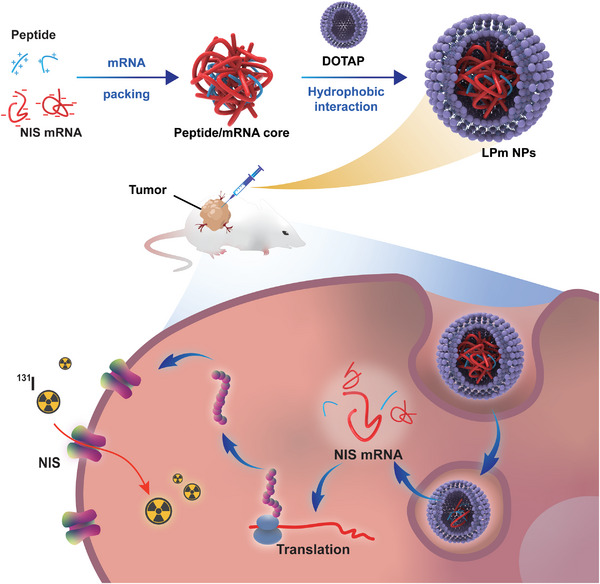
Schematic diagram of the structure of LPm NPs and their function of transporting NIS mRNA in cells for protein expression.

In this project, lipid‐peptide‐mRNA (LPm) nanoparticles (LPm NP) are core–shell nanostructures composed of mRNA, peptide core and cationic liposomes. To achieve high mRNA transfection efficiency, we designed multiple peptide cores for nucleic acid adsorption. Thus, we designed a library containing four arginine‐rich peptides (as listed in **Table** **1**). Protamine, a clinically used arginine‐rich peptide for mRNA condensation was set as control. Nine‐arginine peptides (R9) with different modifications were evaluated. Six‐histidine peptides (H6) were conjugated to R9 to provide protonation prosperity and improve endosome escape. Meanwhile, two amphiphilic peptides stearyl‐R9 (C18‐R9) and cholesterol R9 (Chol‐R9) were also chosen to investigate nucleic acid condensation. The formation of mRNA peptides core is a programmed packaging step as described in **Figure** **2**. First, peptide cores were used to condense mRNA into nanosized complexes (mRNA/peptides complexes). Second, the preformed DOTAP liposome interacted electrostatically with mRNA/peptides complexes, inducing the lipid bilayer to collapse onto the core structure. As a result, spherical LPm NP with a core/membrane structure were formed. Both cationic liposomes and peptides facilitate endosomal escape and the cytosolic release of the cargo.

**Table 1:** Sequence of arginine‐rich peptides

Name	Sequence	*N* ^a)^ [mol]
Protamine	PRRRRSSSRPVRRRRRPRVSRRRRRRGGRRRR	20
R9H6	RRRRRRRRRHHHHHH	9.6
C18‐R9	Stearyl‐RRRRRRRRR	9
Chol‐R9	Cholesterol‐RRRRRRRRR	9

^a)^ *N*: amount of substance.

### 2.3 Formulation Optimization of Peptide Cores

**Figure 3:**
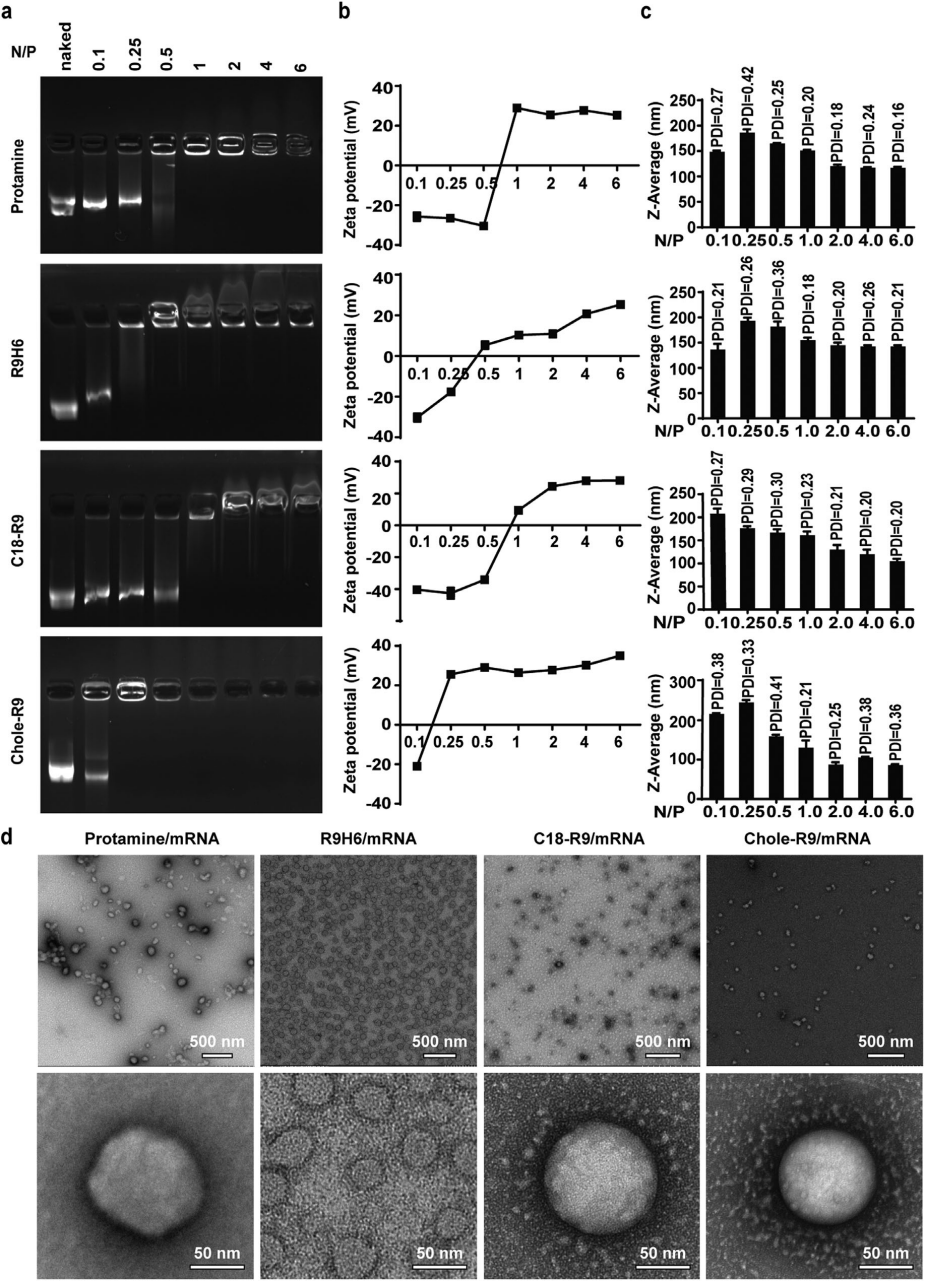
Morphology and stability of peptide/mRNA complexes. a) Agarose gel electrophoresis at different ratios of mRNA and peptides. ZETA potential b) and nanoparticle size c) of different ratios of N/P were confirmed by dynamic light scattering, DLS measurements. d) Peptide/mRNA complexes morphology observed by TEM (scale bars: 500 and 50 nm). N/P: peptides/mRNA.

The nucleic acid condensation capability of peptides was assessed by an electrophoretic mobility shift assay. The ratio between positively charged nitrogen (N) from peptides and negatively charged phosphorus (P) in nucleic acid is decisive for physicochemical particle characteristics and biological activity. The N/P ratio was obtained by calculating the weight that carries a 1 mol unit charge. We used enhanced green fluorescent protein (EGFP) mRNA combined with peptides in different ratios for agarose gel electrophoresis. The complete retardation of the mRNA happened at an N/P = 1 for protamine and C18‐R9, N/P = 0.5 for R9H6, and N/P = 0.25 for Chol‐R9 (**Figure** **3**a), indicating that these peptides could effectively condense nucleic acid at these N/P values and at the N/P larger than 1, there was no leaching of mRNA as shown by electrophoresis. The optimal formulation could be evaluated based on particle size, zeta potential, and in vitro transfection efficiency. The mean hydrodynamic particle size and surface charge of peptide/mRNA complexes were determined using dynamic light scattering (DLS). The peptide/mRNA complexes have positive charge at N/P = 1 (Figure 3b), which formed stable complexes with uniform size distribution (polydispersity index [PDI] < 0.2) (Figure 3c). Transmission electron microscopy (TEM) images showed that the peptide/mRNA complexes self‐assembled into typical spherical structures with diameters of ≈100 nm (Figure 3d). Increasing N/P value led to increasing zeta potential and a decreased particle size. Based on these results, the N/P values 0.5, 1, and 2 were taken into consideration for liposome coating.

### 2.4 In Vitro Transfection Efficiency of LPm NP

**Figure 4:**
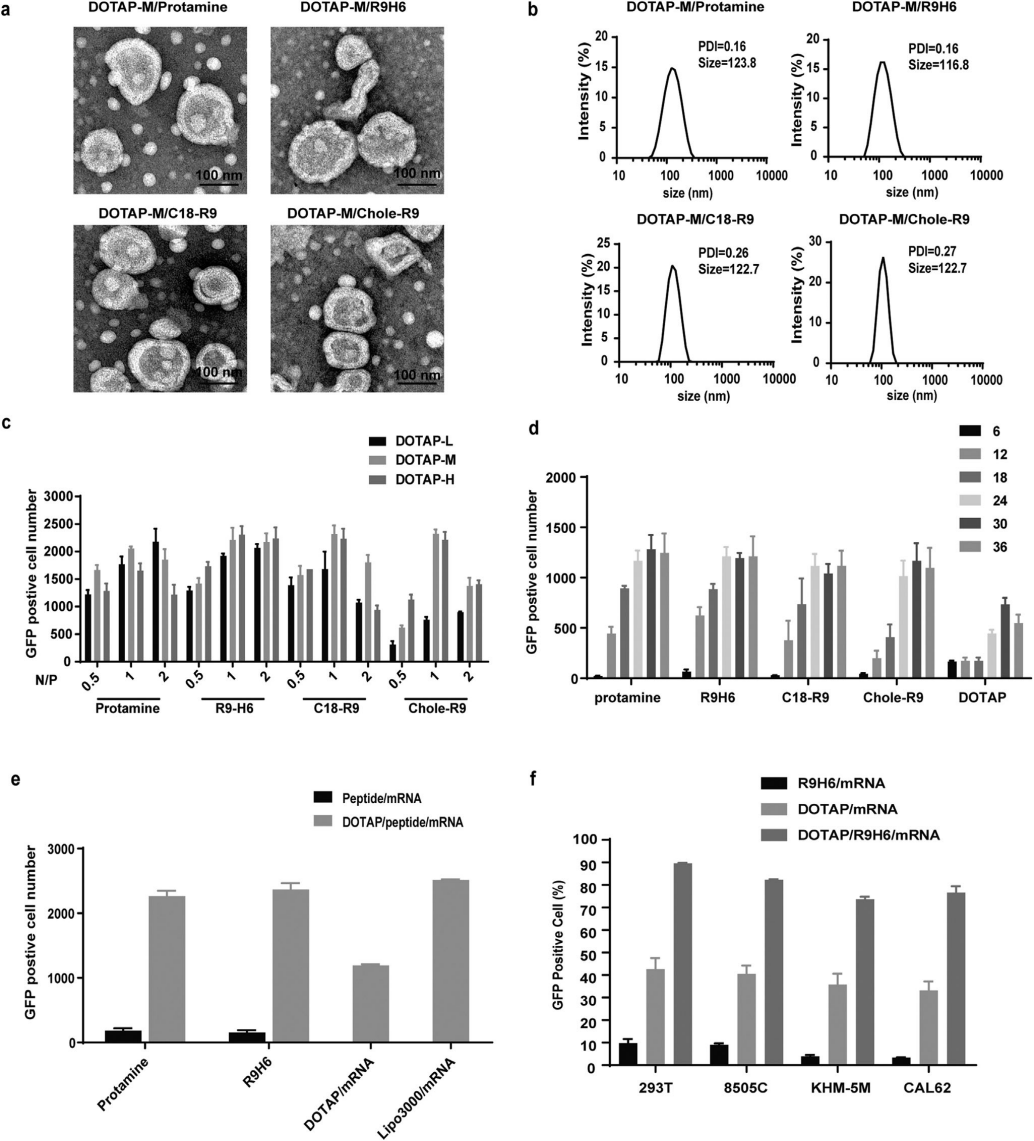
Formulation optimization and morphological characteristics of LPm NPs. a) The morphology of LPm NPs observed by TEM (scale bars: 100 nm). b) Nanoparticle size of LPm NPs with different cores. c) 293T cells were transfected by different lipid‐peptide‐EGFP‐mRNA formulation. After 24 h, GFP‐positive cell number was counted by high‐content imaging systems. L: 12 nmol DOTAP per µg mRNA, M: 24 nmol DOTAP per µg mRNA, H: 36 nmol DOTAP per µg mRNA. d) 293T cells were transfected based on the ratio of 6–36 nmol DOTAP/µg mRNA, and the experimental method was the same as (c). 6:6 nmol DOTAP per µg mRNA, 12:12 nmol DOTAP per µg mRNA, 18:18 nmol DOTAP per µg mRNA, 24:24 nmol DOTAP per µg mRNA, 30:30 nmol DOTAP per µg mRNA, 36:36 nmol DOTAP per µg mRNA. e) Comparison of transfection efficiency of DOTAP cationic liposomes with different cores and Lipofectamine 3000. f) Comparison of transfection efficiencies of different components of LPm NPs in 239T and ATC cells 8505C, KHM‐5 M, and CAL62.

The prepared DOTAP liposomes were divided into three dose groups: DOTAP‐L (12 nmol DOTAP/1 µg mRNA), DOTAP‐M (24 nmol DOTAP/1 µg mRNA) and DOTAP‐H (36 nmol DOTAP/1 µg mRNA). In vitro transfection experiments were performed using these groups in combination with the four core complexes at three sets of N/P ratios. TEM images showed that each group of complexes could self‐assemble into spherical structures with diameters about 120 nm (**Figure** **4**a; Figure S2a, Supporting Information), and the size distribution of the DOTAP‐M group was more uniform (PDI < 0.2) (Figure 4b; Figure S2b, Supporting Information). Then 293T cells were transfected with the prepared LPm NPs nanoparticles in vitro, and the number of GFP‐positive cells was counted by a high throughput imaging system after 24 h incubation. The results indicated that the DOTAP‐M group and DOTAP‐H group showed high transfection efficiency (Figure 4c). These results show that the transfection effect of the experimental group with R9H6 as the core is better than that of other cores, and when the N/P ratio is 1.0, the transfection effect of all experimental groups yielded the best performance.

Next, we continued to optimize the DOTAP liposome ratio using peptide/mRNA complexes with N/P = 1.0 as the core. The peptide/mRNA complexes were combined with 6 different doses of DOTAP liposomes to evaluate their mRNA delivery efficiency in 293T cells. Compared with DOTAP liposomes without a core, DOTAP liposomes with a core showed a sufficiently high delivery effect when the dose was increased to 24 nmol DOTAP/1 µg mRNA (Figure 4d). In addition, the transfection effect of LPm NPs with R9H6 as the core of the vector was the best. Therefore, we selected R9H6 as the lipid core and a DOTAP liposome dose of 24 nmol DOTAP/1 µg mRNA to prepare LPm NPs.

Further EGFP‐mRNA delivery analysis found that the transfection efficiency of LPm NPs (R9H6) was almost the same as protamine‐mRNA liposome and lipo3000‐mRNA (Figure 4e). To verify the transfection efficiency of LPm NPs in human thyroid cancer cell lines, we transfected three types of human thyroid cancer cells and 293T cells for EGFP‐mRNA delivery. The results showed that LPm NPs could deliver EGFP mRNA into these three types of human thyroid cancer cells, and showed higher transfection efficiency in 8505C cells (Figure 4f).

Compared with cationic liposome (DOTAP) or peptide complex, the main advantage of LPm NPs is that it can completely condense and encapsulate mRNA, making the formula more stable. Because in the production process of wrapping mRNA with DOTAP alone, In the production process of wrapping mRNA with DOTAP, mRNA was exposed on the surface of DOTAP, the stability and delivery efficiency of mRNA are affected by nucleases in vivo and in vitro.^[^ ^35^, ^36^ ^]^ In LPm NPs, R9H6 could condense mRNA into solid nuclei (Figure 3d), while liposomes provide lipid bilayer shells to form a core–shell structure (Figure 4a). In the process of transmission, this protection strategy prevents mRNA from being degraded by ribozyme and improves the efficiency of gene expression. The optimal proportion of peptide/mRNA complexes and DOTAP is the key to ensure efficient mRNA delivery and transfection. Therefore, LPm NPs are a good candidate for mRNA delivery with high potential.

### 2.5 Validation of LPm NPs‐Mediated NIS Expression and ^131^I Uptake

**Figure 5:**
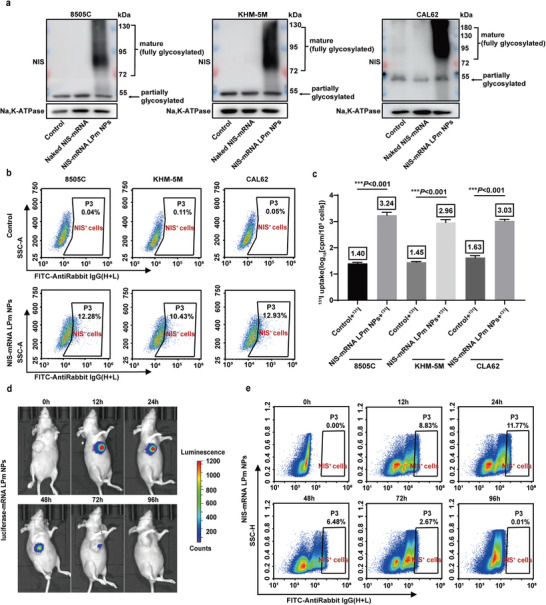
LPm NPs‐mediated protein distribution. a) Western blot analysis on the membrane protein in ATC cell lines after treatment with naked NIS‐mRNA or NIS‐mRNA LPm NPs. b) Flow cytometry on ATC cell lines after NIS‐mRNA LPm NPs treatment. c) Restored ^131^I uptake by ATC cell lines after NIS‐mRNA LPm NPs treatment. d) In vivo imaging of small animals at different time periods after luciferase‐mRNA LPm NPs treatment. e) Flow cytometry results of mouse tumor tissue at different time periods after NIS‐mRNA LPm NPs treatment.

To verify whether the deletion of NIS protein in ATC cell membrane could be restored by NIS‐mRNA delivery using LPm NPs, the three ATC cell types were incubated with PBS (negative control), naked NIS‐mRNA, and NIS‐mRNA LPm NPs. After 24 h, the membrane proteins of the cells in each group were extracted and verified by Western blotting (WB). The NIS‐mRNA LPm NPs group had an obvious fully glycosylated band of NIS protein (**Figure** **5**a; Figure S3, Supporting Information), while the cells treated with PBS and naked NIS‐mRNA did not. Deletion or reduced expression of fully glycosylated NIS protein resulted in resistance to radioactive iodine therapy in ATC cells.^[^ ^37^, ^38^, ^39^ ^]^ The expression of NIS proteins in ATC cells after NIS‐mRNA LPm NPs treatment was further evaluated. Flow cytometric analysis was carried out and the results showed that the proportion of NIS^+^ cells in ATC cells treated with NIS‐mRNA LPm NPs was significantly increased compared with the control group 24 h after transfection (Figure 5b; Figure S4a, Supporting Information), and the proportion of NIS^+^ cells in 8505C cells increased to about 13% on average (Figure S5a, Supporting Information).

To verify whether the NIS proteins expressed in the cell membrane have the function of iodine uptake, we performed ^131^I uptake experiments on three ATC cell types. We began by allowing 1µCi ^131^I to incubate for 2 h, washed twice with precooled PBS, collected, and counted the cells, and measured the radioactivity of the cells with a gamma counter to evaluate the amount of ^131^I uptake. The iodine uptake of the three cell lines was significantly increased (Figure 5c) (*p* < 0.001), of which 8505C yielded the best effect, with a 70‐fold increase. Subsequently, 8505C cells were divided into 5 groups to verify the iodine intake of different systems: PBS group, ^131^I group, EGFP‐mRNA LPm NPs combined with ^131^I group, empty LPm NPs combined with ^131^I group, and NIS‐mRNA LPm NPs combined with ^131^I group. Gamma counter detection was performed after corresponding treatment, and the results showed that only NIS‐mRNA LPm NPs could increase cellular iodine uptake (Figure S5b, Supporting Information) (*p* < 0.001). These results indicate that LPm NPs can efficiently deliver NIS‐mRNA into cells, increase the level of complete glycosylation of NIS membrane proteins, and restore the ability of ATC cells to take up radioiodine.

After intratumoral administration of luciferase‐mRNA LPm NPs in 8505C tumor‐bearing nude mice, the expression of luciferase was examined using a small animal imaging system. It was showed that the fluorescence signal was the highest between 12 and 24 h, and could be maintained for about 72 h after administration of the luciferase‐mRNA LPm NPs (Figure 5d; Figure S5c, Supporting Information). Intratumoral injection of NIS‐mRNA LPm NPs was performed every 3d to further evaluate the expression of NIS protein in the tumor of mice. Tumor tissues from mice (*n* = 3) were harvested at 0, 12, 24, 36, 48, 72, and 96 h after administration, dissociated into single cells, and the NIS^+^ cells were detected by flow cytometry (see Figure S4b in the Supporting Information for flow gating strategy). The results showed that NIS protein expression peaked between 12 and 24 h and then gradually decreased (Figure 5e; Figure S5d, Supporting Information), and NIS expression was maintained for about 72 h. These results indicated that after NIS‐mRNA LPm NPs were delivered in vivo, the proportion of NIS protein‐positive cells was highest after 12–24 h, and almost extinguished at 72–96 h. Therefore, the administration frequency of NIS‐mRNA LPm NPs was set as once every 3 days, and the administration time of ^131^I was set at 12 h after administration of NIS‐mRNA LPm NPs.

After intratumoral injection of NIS mRNA, we further compared the expression of NIS on tumor cells, fibroblasts and macrophages (Figure S6, Supporting Information), The results showed that different cell populations in the tumor had no specific selection for LPm NPs uptake. In tumor tissue, except for tumor cells, other cells will also absorb LPm NPs and express NIS. When combined with ^131^I therapy, these cells will also be damaged by radiation. Cancer associated fibroblasts (CAF) and tumor‐associated macrophage (TAM) are the main cell groups in all solid tumor stroma. They usually play a role in promoting tumor formation, promoting tumor progression and resistance to treatment, and may play a synergistic role.^[^ ^40^, ^41^ ^]^ The killing of CAF and TAM is also conducive to tumor treatment.

### 2.6 Intratumoral Injection of NIS‐mRNA LPm NPs Leads to Local Expression of NIS Enables ^131^I Uptake

**Figure 6:**
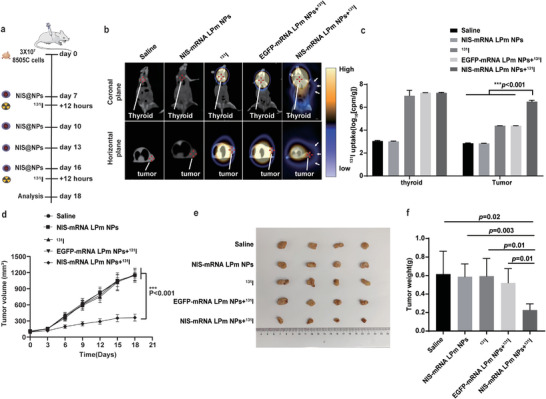
Intratumoral injection of NIS‐mRNA LPm NPs leads to local expression of NIS and enables ^131^I uptake. a) Schematic diagram of the combined administration procedure of NIS‐mRNA and ^131^I based on LPm NPs. b) SPECT/CT imaging of mice treated with saline, NIS‐mRNA LPm NPs, ^131^I, EGFP‐mRNA LPm NPs + ^131^I, or NIS‐mRNA LPm NPs + ^131^I for 24 h. The radioactivity count of ^131^I was detected by a gamma counter and plotted in (c). d) Tumor growth curves after subcutaneous implantation of 8505C tumor cells in mice, recorded every three days (*n* = 4). Image (e) and tumor weight (f) of the excised subcutaneous tumor on day 18.

To test whether NIS‐mRNA LPm NPs could restore iodine uptake in ATC tumor tissue and achieve therapeutic effects, we used the 8505C cell line to construct a mouse ATC subcutaneous tumor model for ^131^I combined treatment experiments. The treatment procedure is shown in **Figure** **6**a. We randomly divided the mice into 5 groups: saline group, NIS‐mRNA LPm NPs group, ^131^I group, EGFP‐mRNA LPm NPs + ^131^I group, and NIS‐mRNA LPm NPs + ^131^I group. 24 h after the corresponding treatment in each group, the distribution of ^131^I in vivo was imaged by single‐photon emission computed tomography/x‐ray computed tomography (SPECT/CT). Only mice in the NIS‐mRNA LPm NPs combined with ^131^I treatment group had obvious ^131^I accumulation in tumor sites, and normal iodine accumulation in their thyroid sites (which normally express NIS protein) was seen (Figure 6b). The rest of the experimental groups that were administered ^131^I only had ^131^I radioactive signals in the thyroid, and no radioactive signals were found in the whole body of the mice that were not given ^131^I treatment.

Mice in each experimental group were randomly selected based on the imaging results. The tumor tissue and thyroid tissue with obvious ^131^I imaging were surgically removed, and the radioactivity of the tissue was assessed using a gamma counter. The radioactivity of tumor tissue in the NIS‐mRNA LPm NPs+^131^I group was significantly higher than that in the other ^131^I‐treated groups by more than 4000 times (Figure 6c) (*p* < 0.001). There was no significant difference in the iodine uptake rate of thyroid tissue between all groups treated with ^131^I. This suggests that NIS‐mRNA LPm NPs can restore the expression of NIS protein in the ATC mouse model, thereby reactivating the uptake of ^131^I in the lesions. Both tumor growth curve and tumor weight statistics showed that, compared with other control treatments, the tumor growth was significantly suppressed in the experimental group treated with ^131^I combined with NIS‐mRNA LPm NPs (Figure 6d–f).

NIS protein is a recognized as an essential protein for ^131^I therapy. We successfully reversed the iodine insensitivity of ATC by introducing NIS‐mRNA LPm NPs into mouse ATC tumor tissue. To verify the safety of this method in vivo, subcutaneous tumor‐bearing healthy mice treated with local injection of NIS‐mRNA LPm NPs were euthanized and major organs (heart, liver, spleen, lung and kidney) were harvested for hematoxylin and eosin (H&E) staining. No significant tissue damage (Figure S7, Supporting Information) was found in mice treated with NIS‐mRNA LPm NPs.

Emerging nanotechnology provides an opportunity to improve the cytoplasmic delivery of different mRNA therapeutic drugs into cancer cells. Some NPs mediated mRNA drug delivery has entered the clinical trial stage.^[^ ^42^, ^43^ ^]^ The key to success in the field of mRNA drugs is to be able to enter cells for stable and efficient transfection. Among them, lipid carriers are the most widely studied.^[^ ^44^ ^]^ In order to better overcome the shortcomings of poor stability and harsh storage conditions of liposome carriers used alone, adding cores in liposomes is a common method at present, which can improve the stability of carriers and optimize the storage conditions of nucleic acids.^[^ ^45^, ^46^ ^]^ LPm NPs are designed and improved on this basis, which can deliver NIS mRNA more stably and efficiently. Polymers and dendritic cells are also widely used for mRNA delivery, showing good stability and delivery efficiency, but they are not widely used due to their toxicity or complex in vitro culture process. Due to the poor stability of mRNA in solution and in vivo, the effect of mRNA therapy can be further improved. For example, the development of functionalized mRNA to improve stability and expression can maximize the impact of therapeutic mRNA production, transmission and management, but the redesigned and modified mRNA is also a challenge to intracellular translation and protein output.^[^ ^47^, ^48^ ^]^ In the future, our further research may need to focus on the structural modification of NIS mRNA and its combination with other auxiliary materials to further improve the stability and transfection efficiency of NIS mRNA, increase the targeting of ATC cells, while not sacrificing the drug delivery effect.

## 3 Conclusion

In summary, we not only provided evidence for restoring NIS expression in ATC through mRNA strategy, but also developed an efficient NP platform for transfection, which is used for in situ transfection of mRNA in ATC tumors. This study designed multiple peptide core for mRNA adsorption based on the principle of amphiphilic polypeptide assembly, and the resulting LPm NPs nanoparticles may be applicable to mRNA therapy. The components of LPm NPs are safe and simple to synthesize, and can efficiently deliver mRNA into tumor cells. NIS‐mRNA delivery with LPm NPs can restore the expression of NIS protein in ATC cell membrane, and thus improve the iodine uptake by ATC when combined with ^131^I treatment. The strategy proposed in this study may develop into a new adjuvant therapy for thyroid cancer in clinic to convert non resectable ATC primary tumors into resectable tumors.

## 4 Experimental Section

### Cell Culture

Cell lines 8505C (DSMZ, ACC‐219), KHM‐5 M (Expasy, CVCL_2975), and CAL62 (DSMZ, ACC‐448) were cultured with 10% fetal bovine serum (30044333, Gibco, USA), 100 units mL^−1^ penicillin and 100 µg mL^−1^ streptomycin (450‐201‐CL, WISENT, Canada) in RPMI‐1640 (11879020, Gibco, USA) medium. Cell line 293T (ATCC, CRL‐3216) was incubated with 10% fetal bovine serum (30044333, Gibco, USA), 100 units mL^−1^ penicillin and 100 µg mL^−1^ streptomycin (450‐201‐CL, WISENT, Canada) in DMEM (A1896701, Gibco, USA).

### mRNA Synthesis

mRNA was synthesized by in vitro transcription (IVT) using the HiScribe T7 ARCA mRNA Kit (with tailing) (E2060S, NEB, USA). The template for IVT was the PCR product from plasmid pST1374 using Q5 High Fidelity 2X Master Mix (M0492, NEB, USA). The template contains the T7 promoter and the target sequence is provided in Table S1 (Supporting Information). The IVT reaction was then carried out according to the standard protocol, except that CTP and UTP were replaced by 5‐methyl‐CTP and pseudo‐UTP, respectively. Finally, mRNA was 3′‐poly(A)‐tailed using poly(A) polymerase (M0276L, NEB, USA), and mRNA purification was achieved by LiCl precipitation according to the manufacturer's instructions.

### Construction of mRNA‐LPm NPs

Protamine (P4005‐250MG) was purchased from Sigma Aldrich (USA). Anhydrous dextrose (DGC509‐1KG) was purchased from Dogesce (China). The based amphiphilic polypeptides R9H6, Chol‐R9 and C18‐R9 were synthesized by Fmoc solid‐phase peptide synthesis by China Peptides CO. Ltd. (Shanghai, China). DOTAP (O02002, AVT, China) and cholesterol (O01001, AVT, China) were dispersed in 2 mL of dichloromethane in a round glass flask at a molar ratio of 1:1. The mixture was then rotary evaporated at 36 °C for 30 min to form a lipid film. After hydration with 1 mL of DPEC treatment water, it was extruded through a 100 nm/200 nm extruder. Using 5% glucose as the solvent, mRNA‐polypeptide nanoparticles were prepared according to the experimental ratio, and then combined with DOTAP liposomes.

### DOTAP Liposome Ratio Optimization

In the initial optimization, the prepared DOTAP liposomes were divided into DOTAP‐L (12 nmol DOTAP per 1 µg mRNA), DOTAP‐M (24 nmol DOTAP per 1 µg mRNA), and DOTAP‐H (36 nmol DOTAP per 1 µg mRNA) The three dose groups were combined with the four peptide/mRNA complexes in the N/P ratios of the three groups. 293T cells were seeded into 96‐well plates, and treated according to different experimental groups for 24 h. Cells were imaged and analyzed by a high‐content cell analyzer (ArrayScan VTI, Thermo Fisher, USA).

For further optimization, 6 groups of different DOTAP liposomes combined with polypeptides core‐mRNA core with an N/P ratio of 1.0 were designed according to the dose of 6–36 nmol DOTAP/1 µg mRNA, and the 293T cells were further transfected. The specific groups were: 6 nmol DOTAP per µg mRNA, 12 nmol DOTAP per µg mRNA, 18 nmol DOTAP per µg mRNA, 24 nmol DOTAP per µg mRNA, 30 nmol DOTAP per µg mRNA, and 36 nmol DOTAP per µg mRNA.

### Electrophoretic Mobility Shift Assay

To test the mRNA concentration ability of peptides, 2 µg of mRNA was incubated with various amounts of peptides in 10 µL of phosphate‐buffered saline (PBS) (20012027, Gibco, USA) for 20 min at room temperature. Peptide/mRNA complexes’ N/P ratio ranges from 1:10 to 6:1. Peptide/mRNA complexes were then analyzed by 1% agarose gel electrophoresis in TAE (40 × 10^−3^ m Tris acetate/2 × 10^−3^ m EDTA) buffer at 120 V for 10 min. The resulting gel was then stained with SYBR GREEN II (Invitrogen, USA) for 5 min and visualized on a ChemiDoc Touch Imaging System (Bio‐Rad, USA).

### Characterization of Nanoparticles

The morphology and size distribution of nanoparticles were characterized by TEM (Ht‐7700, Hitachi, Japan) using phosphotungstic acid (Zhongjingkeyi Technology, China) negative staining method. Size distribution (diameter, nm) and surface charge (zeta potential, mV) were determined using a ZetaSizer Nano series Nano‐ZS (Zetasizer Nano ZS90, Malvern, UK) equipped with a He‐Ne laser beam with a wavelength of 633 nm and a 90° fixed scattering angle. Assays were performed on appropriately diluted samples in 10 × 10^−3^ m HEPES buffer at room temperature.

### Western Blot Analysis

Cells were seeded into 6‐well plates. Membrane proteins were extracted 24 h after transfection using a membrane protein extraction kit (A10008S, Abmart, China). The extract was supplemented with 1.0 × 10^−3^ m phenylmethyl sulfonyl fluoride (PMSF) (Solarbio, China) in RIPA buffer. The concentration of membrane proteins was quantified by BCA assay kit (Thermo Pierce Chemical, USA). They were separated by SDS‐PAGE electrophoresis and then transferred to polyvinylidene fluoride (PVDF) membranes. These membranes were blocked with 5% bovine serum albumin (Solarbio, China) for 1 h and incubated with primary antibodies overnight at 4 °C. Subsequently, the membrane was washed 3 times with TBST (Tris‐buffered saline, 0.1% Tween 20) and incubated with secondary antibody for 1 h. Immunoreactivity was visualized and scanned under a chemiluminescence imaging system by enhanced chemiluminescence reagents (Thermo Scientific, USA). The following primary antibodies were prepared: Rabbit anti‐NIS (1:1000) (24324‐1‐AP, Proteintech, USA) and rabbit anti‐Na+/K+‐ATPase (1:1000) (ABP51894, Abbkine, China). Secondary antibody was Horseradish peroxidase (HRP)‐conjugated goat anti‐rabbit IgG (1:10 000) (Santa Cruz, USA, sc‐2004).

### Animal Experiment

All animal protocols were approved by the Ethics Committee of the Ethics Committee of the Cancer Hospital Affiliated to the University of Chinese Academy of Sciences (The approval number of the animal experiment is 2021‐10‐001). Female, 6‐week‐old, BALB/c‐nu mice were purchased from Viton Lever (Beijing, China).

### Tumor Inoculation and Tumor Treatment

The ratio of sterile PBS to Matrigel (354 230, CORNING, USA) was 1:1 and the volume was 100 µL, containing 3 × 10^6^ 8505C cells for subcutaneous injection. After 7–9 days, when the tumor size reached 50 mm^2^, the mice were randomly divided into 5 groups (*n* = 8). Each group was independently processed with intratumoral injection volume of 50 µL, mRNA dose of 350 µg kg^−1^.

### Flow Cytometry

1) The expression of NIS in cell lines was analyzed by flow cytometry. 24 h after the administration of NIS‐mRNA LPm NPs to the cell lines, the cells were trypsinized and resuspended, and the blank treatment group was used as a control. Cells were resuspended in 1640 medium (20012027, Gibco, USA) containing 3% FBS. Cells were incubated with rabbit anti‐NIS (1:1000) (24324‐1‐AP, Proteintech, USA) at 4 °C for 1.5 h, washed with PBS, and incubated with goat anti‐rabbit IgG (1:300) conjugated to coralite488 (SA00013‐2, Proteintech, USA) for 1.5 h at 4 °C. Staining was terminated with PBS, and cells were washed and resuspended in 200 µL of 1640 medium for analysis on an ACEA NovoCyte flow cytometer (ACEA NovoCyte, USA). The proportion of positive cells was analyzed by appropriate gating to exclude cell aggregates and debris from the analysis. 2) The expression of NIS on the cell surface of tumor tissue was analyzed by flow cytometry. ATC subcutaneous tumor mice were randomly divided into 6 groups (*n* = 3) according to the detection time of 0, 12, 24, 48, 72, and 96 h after administration. Mouse tumors were surgically removed. Preparation of tumor tissue lysate was performed using 7.5 mg collagenase (17104019, Gibco, USA) and 0.15 mg DNase (D806930, Macklin, China) diluted to 15 mL in serum‐free DMEM. Different parts of the tumor tissue were put into 1.5 mL EP tubes, lysis buffer was added, and centrifuged at 150 rpm, 37 °C for 30 min, and then centrifuged to obtain tumor cell suspension. The subsequent staining method and on‐machine analysis method are the same as (1). 3) The expression of NIS on 8505C cells, tumor associated fibroblasts (CAF) and tumor associated macrophages (TAM) was detected by flow cytometry. The method of obtaining cell suspension is the same as that of NIS staining (2). Antibodies used for different cell populations are as follows: PE CD147 (306212, USA), Anti alpha smooth muscle Action/APC (bs‐0189R‐APC, China), PE cy7 F480 (14‐4801‐82, USA).

### In Vivo Imaging of Small Animals

Mice were grouped Randomly (*n* ≥ 3) after intratumoral injection of luciferase‐mRNA LPm NPs in batches. Small animal live imaging was performed at different time points, and D‐luciferin potassium salt (7903‐100, Biovision) was prepared with sterile PBS working solution (15 mg mL^−1^), and sterilized with a 0.2 µm filter. The natural substrate of luciferase was intraperitoneally injected into groups according to different time periods, where a dose of 10 µL g^−1^ and a 150 mg/kg luciferin working solution was administered. Imaging analysis was performed after 10–15 min using a small animal in vivo imaging system (IVIS Spectrum, USA) (*n* = 3).

### 4.1 Treatment and Testing

^131^I treatment was administered 12 h after NIS‐mRNA LPm NPs administration. The cells were seeded into a 6‐well plate, incubated with 1µCi ^131^I for 2 h, washed twice with precooled PBS, digested with trypsin, collected and counted, and the radioactivity of the cells was detected by a gamma radioimmunoassay (GC‐1500, ZONKIA, China) to assess radioactive iodine uptake. 12 h after intratumoral LPm NP administration, and 24 h after intraperitoneal injection of 1 mCi ^131^I, the distribution of ^131^I in mice in different treatment groups were imaged by SPECT /CT (Discovery NMCT 670, GE, USA). SPECT /CT showed that thyroid tissue and tumor tissue were the main accumulation sites of ^131^I in mice. Tumor and thyroid tissue of mice in different experimental groups (*n* = 4) were randomly dissected, and the radioactivity of the tissue was detected and counted by *γ*‐radioimmunoassay.

### Participant Cohort

1) The use of human samples was approved by the Ethics Committee of Cancer Hospital Affiliated to the University of Chinese Academy of Sciences (IRB‐2019‐8). Informed consents were obtained from all subjects. Patients diagnosed with thyroid cancer after histological and cytological examinations who had not been previously treated were eligible for inclusion in the study. Protein expression levels were determined by immunohistochemical (IHC) staining of 111 thyroid cancer patient samples using rabbit anti‐NIS (24324‐1‐AP, Proteintech, USA). IHC slides were graded independently by two pathologists blinded to patient outcomes. Discordant cases were evaluated by a third pathologist and a consensus was reached. Semiquantitative scoring of NIS immunoreactivity was based on the intensity and extent of tumor cell staining.^[^ ^49^ ^]^ 2) Data on patients with thyroid cancer who were treated for the first time in Zhejiang Cancer Hospital and received surgical treatment from January 2016 to December 2017 were collected, with a follow‐up period of 45–68 months. 3) The network database was used to analyze the difference in SLC5A5 expression among histological types of thyroid cancer tissues and its relationship with prognosis. The original data of SLC5A5 expression came from UALCAN (http://ualcan.path.uab.edu/index.html), and the original data for Kaplan‐Meier analysis of recurrence‐free survival came from the cBioPortal database (https://www.cbioportal.org/), in which the top 50% of SLC5A5 were highly expressed and the rest were moderately or lowly expressed.

### Statistical Analysis

Data are presented as mean ± standard deviation (SD). GraphPad Prism software was used to performed multiple comparisons using one‐way analysis of variance (ANOVA) with Tukey's multiple comparison test. Statistical significance was set as follows: *P < 0.05, **P < 0.01, ***P < 0.001, NS means no significant difference.

## Conflict of Interest

The authors declare no conflict of interest.

## Author Contributions

Q.L., L.Z., and J.L. contributed equally to this work. M.G., X.Z., and K.C., designed the research. Q.L., L.Z., J.L., Z. T., Q. F., Q.F., F.Z., G.L., Z.Y., X.Y., H.F., H.Y., Q.W., and T.J. performed the research. All authors analyzed and interpreted the data. Q.L., L.Z., K.C., and J.L. wrote the paper.

## Supporting information

Supporting information

## Data Availability

The data that support the findings of this study are available from the corresponding author upon reasonable request.
