# Supplementary material for: Lipid‐Peptide‐mRNA Nanoparticles Augment Radioiodine Uptake in Anaplastic Thyroid Cancer
Source: Adv Sci (Weinh). 2022 Dec 1;10(3):2204334. doi: 10.1002/advs.202204334 (PMC9875617; doi:10.1002/advs.202204334)
Supplement: Supplementary file 1 — Supporting information [file ADVS-10-2204334-s001.pdf]

ADVANCED SCIENCE
Open Access

Supporting Information

for *Adv. Sci.*, DOI 10.1002/advs.202204334

Lipid-Peptide-mRNA Nanoparticles Augment Radioiodine Uptake in Anaplastic Thyroid Cancer

Qinglin Li, Lizhuo Zhang, Jiayan Lang, Zhuo Tan, Qingqing Feng, Fei Zhu, Guangna Liu, Zhangguo Ying, Xuefei Yu, He Feng, Heqing Yi, Qingliang Wen, Tiefeng Jin, Keman Cheng, Xiao Zhao* and Minghua Ge**

Supplementary Information.

Lipid-Peptide-mRNA Nanoparticles Augment Radioiodine Uptake in Anaplastic Thyroid Cancer

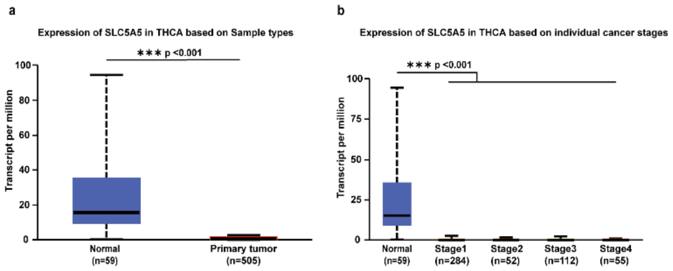

Fig. S1. (a). Expression of SLC5A5 in TC based on Sample types (from UALCAN). (b). Expression of SLC5A5 in TC based on individual cancer stages (from UALCAN). Statistical significance was tested by two-tailed Student's t-test.

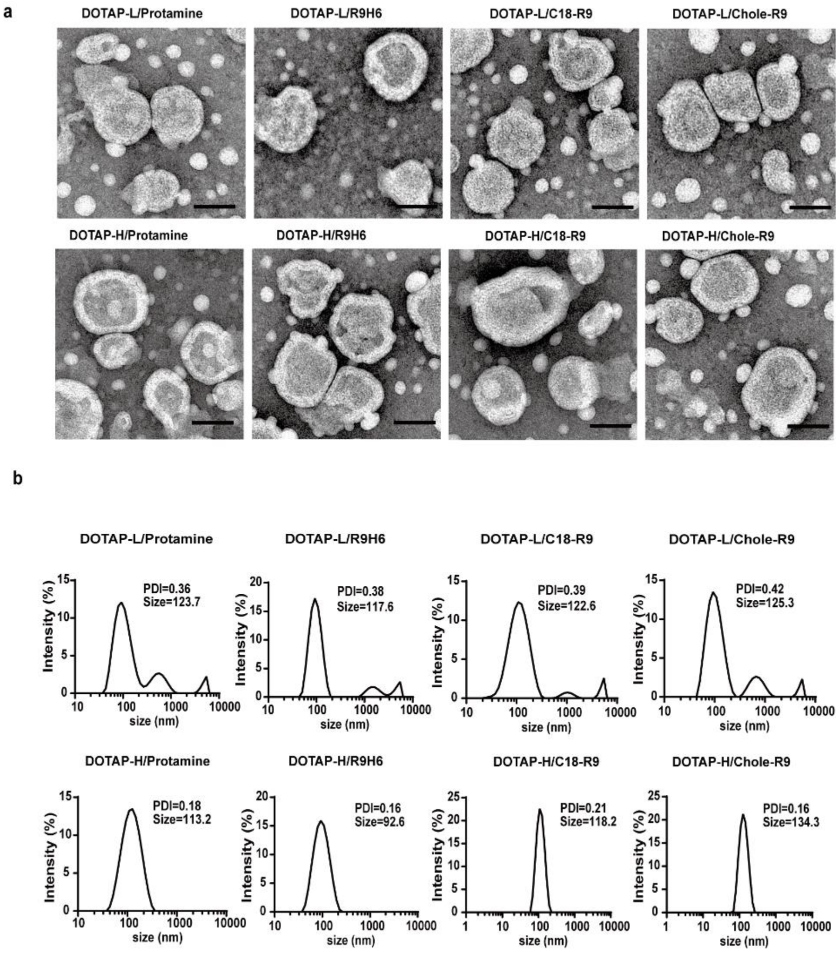

Fig. S2. (a) The morphology of LPm NPs was observed by TEM (Scale bars: 100 nm). (b) Size distribution of LPm NPs with different cores.

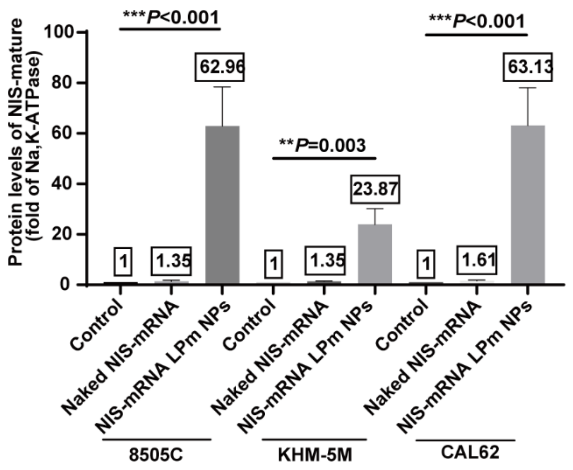

Fig. S3. Quantitative analysis results of fully glycosylated fractions in WB bands; calculated by Image J software by normalizing the intensity of each group of NIS protein bands to the intensities of membrane protein internal reference Na, K-ATPase (n=3 for each group)

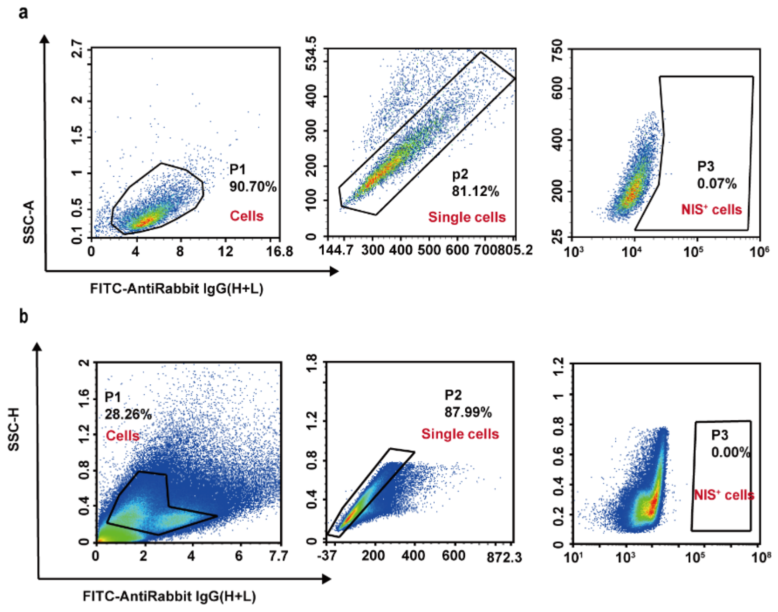

Fig. S4. Flow Cytometry Gating Strategy for analysis of NIS expression *in vitro* (a) and *in vivo* (b).

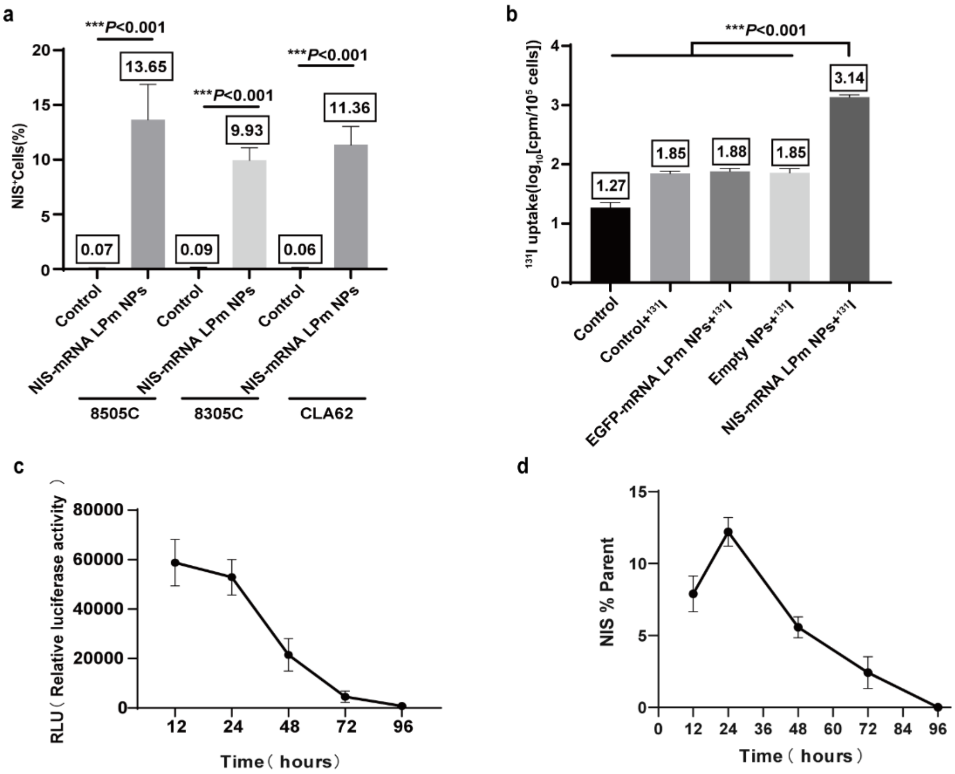

Fig. S5. (a) Statistical analysis of flow cytometry results. (b) Detecting the radioactivity of 8505C cells after different grouping by gamma counter. (c) Quantification curve of fluorescence intensity time gradient for in vivo imaging of small animals (n=3). (d) Quantitative analysis of tumor tissue flow cytometry results (n=3).

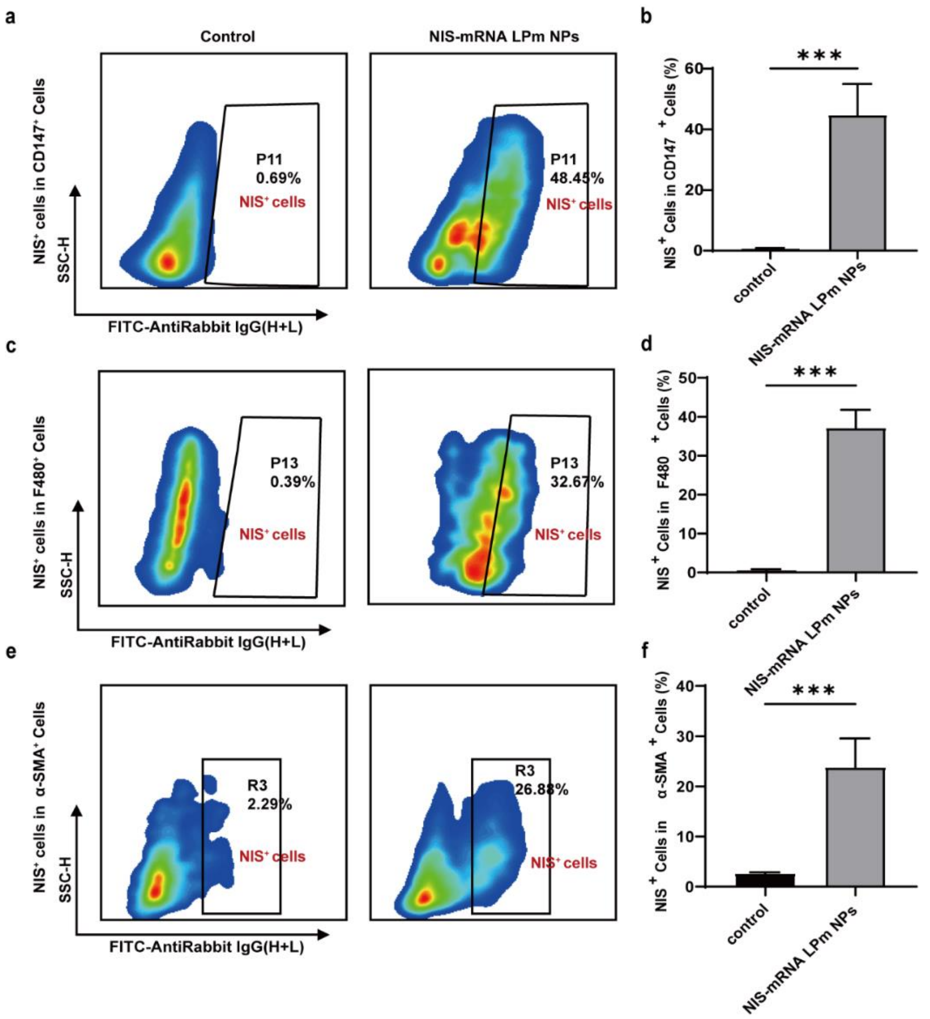

Fig. S6. The expression of NIS on 8505C cells, tumor associated fibroblasts (CAF) and tumor associated macrophages (TAM) was detected by flow cytometry. (a, c, e) NIS+ cells proportion of different cell populations in mouse tumor tissue after NIS-mRNA LPm NPs treatment. (b, d, f) Statistical analysis of flow cytometry results.

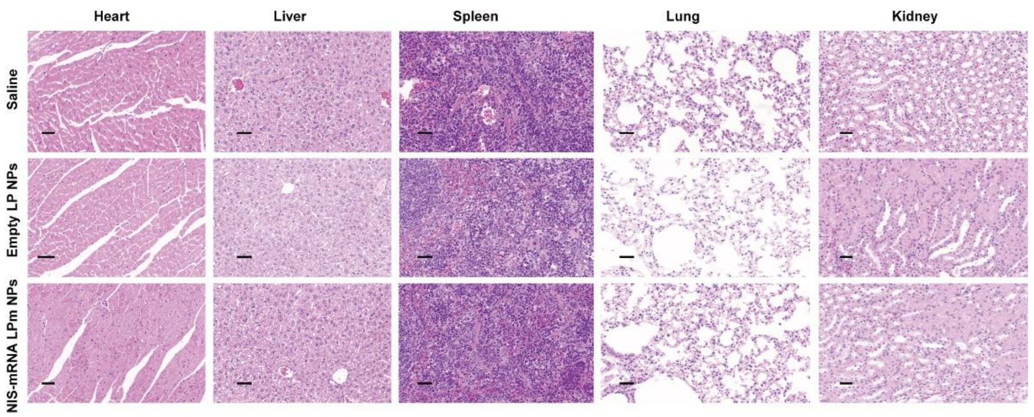

Fig S7. *In vivo* toxicity of the NIS-mRNA LPm NPs strategy via local delivery. Histopathological and hematological (H&E) analysis of the sections for the major organs was performed after intravesical perfusion of PBS, empty LP NPs, or NIS-mRNA LPm NPs. (Scale bar: 40 μm)

Supplementary Table 1. mRNA sequences used in this study.

mRNAs	Sequences
5’-UTR	GGGAAAUAAGAGAGAAAAGAAGAGUAAGAAGAAAUAUAAGAGC<br>CACC
3’-UTR	GCUCGCUUUCUUGCUGUCCAAUUUCUAUUAAAGGUUCCUUUGUU<br>CCCUAAGUCCAACUACUAAACUGGGGGAUAUUAUGAAGGGCCUU<br>GAGCAUCUGGAUUCUGCCUAAUAAAAAACAUUUAUUUUCAUUG<br>CGCUCGCUUUCUUGCUGUCCAAUUUCUAUUAAAGGUUCCUUUGU<br>UCCCUAAGUCCAACUACUAAACUGGGGGAUAUUAUGAAGGGCCU<br>UGAGCAUCUGGAUUCUGCCUAAUAAAAAACAUUUAUUUUCAUU<br>GC
SLC5A5(NIS)	ATGGAGGCCGTGGAGACCGGGGAACGGCCCACCTTCGGAGCCTGG<br>GACTACGGGGTCTTTGCCCTCATGCTCCTGGTGTCCACTGGCATCG<br>GGCTGTGGGTCGGGCTGGCTCGGGGCGGGCAGCGCAGCGCTGAGG<br>ACTTCTTCACCGGGGGCCGGCGCCTGGCGGCCCTGCCCGTGGGCC<br>TGTCGCTGTCTGCCAGCTTCATGTCGGCCGTGCAGGTGCTGGGCGT<br>GCCGTCGGAGGCCTATCGCTATGGCCTCAAGTTCCTCTGGATGTGC<br>CTGGGCCAGCTTCTGAACTCGGTCCTCACCGCCCTGCTCTTCATGC<br>CCGTCTTCTACCGCCTGGGCCTCACCAGCACCTACGAGTACCTGGA<br>GATGCGCTTCAGCCGCGCAGTGCGGCTCTGCGGGACTTTGCAGTAC<br>ATTGTAGCCACGATGCTGTACACCGGCATCGTAATCTACGCACCGGC<br>CCTCATCCTGAACCAAGTGACCGGGCTGGACATCTGGGCGTCGCTC<br>CTGTCCACCGGAATTATCTGCACCTTCTACACGGCTGTGGGCGGCA<br>TGAAGGCTGTGGTCTGGACTGATGTGTTCCAGGTCGTGGTGATGCT<br>AAGTGGCTTCTGGGTTGTCCTGGCACGCGGTGTCATGCTTGTGGGC<br>GGGCCCCGCCAGGTGCTCACGCTGGCCCAGAACCACTCCCGGATC<br>AACCTCATGGACTTTAACCCTGACCCGAGGAGCCGCTATACATTCT<br>GGACTTTTGTGGTGGGTGGCACGTTGGTGTGGCTCTCCATGTATGG<br>CGTGAACCAGGCGCAGGTGCAGCGCTACGTGGCTTGCCGCACAGA

	GAAGCAGGCCAAGCTGGCCCTGCTCATCAACCAGGTCGGCCTGTT<br>CCTGATCGTGTCCAGCGCTGCCTGCTGTGGCATCGTCATGTTTGTGT<br>TCTACACTGACTGCGACCCTCTCCTCCTGGGGCGCATCTCTGCCCC<br>AGACCAGTACATGCCTCTGCTGGTGCTGGACATCTTCGAAGATCTG<br>CCTGGAGTCCCCGGGCTTTTCCTGGCCTGTGCTTACAGTGGCACCC<br>TCAGCACAGCATCCACCAGCATCAATGCTATGGCTGCAGTCACTGT<br>AGAAGACCTCATCAAACCTCGGCTGCGGAGCCTGGCACCCAGGAA<br>ACTCGTGATTATCTCCAAGGGGCTCTCACTCATCTACGGATCGGCCT<br>GTCTCACCGTGGCAGCCCTGTCCTCACTGCTCGGAGGAGGTGTCCT<br>TCAGGGCTCCTTCACCGTCATGGGAGTCATCAGCGGCCCCCTGCTG<br>GGAGCCTTCATCTTGGGAATGTTCCTGCCGGCCTGCAACACACCGG<br>GCGTCCTCGCGGGACTAGGCGCGGGCTTGGCGCTGTCGCTGTGGG<br>TGGCCTTGGGCGCCACGCTGTACCCACCCAGCGAGCAGACCATGA<br>GGGTCCTGCCATCGTCGGCTGCCCGCTGCGTGGCTCTCTCAGTCAA<br>CGCCTCTGGCCTCCTGGACCCGGCTCTCCTCCCTGCTAACGACTCC<br>AGCAGGGCCCCCAGCTCAGGAATGGACGCCAGCCGACCCGCCTTA<br>GCTGACAGCTTCTATGCCATCTCCTATCTCTATTACGGTGCCCTGGG<br>CACGCTGACCACTGTGCTGTGCGGAGCCCTCATCAGCTGCCTGACA<br>GGCCCCACCAAGCGCAGCACCCTGGCCCCGGGATTGTTGTGGTGG<br>GACCTCGCACGGCAGACAGCATCAGTGGCCCCCAAGGAAGAAGTG<br>GCCATCCTGGATGACAACTTGGTCAAGGGTCCTGAAGAACTCCCCA<br>CTGGAAACAAGAAGCCCCCTGGCTTCCTGCCCACCAATGAGGATC<br>GTCTGTTTTTCTTGGGGCAGAAGGAGCTGGAGGGGGCTGGCTCTT<br>GGACCCCCTGTGTTGGACATGATGGTGGTCGAGACCAGCAGGAGA<br>CAAACCTCGACTACAAAGACGACGACGACAAAGACTATAAAGATG<br>ACGACGATAAAGACTACAAGGATGACGATGATAAATGA
EGFP	AUGGUGAGCAAGGGCGAGGAGCUGUUCACCGGGGUGGUGCCCA<br>UCCUGGUCGAGCUGGACGGCGACGUAAACGGCCACAAGUUCAGC<br>GUGUCCGGCGAGGGCGAGGGCGAUGCCACCUACGGCAAGCUGAC<br>CCUGAAGUUCAUCUGCACCACCGGCAAGCUGCCCGUGCCCUGGC<br>CCACCCUCGUGACCACCCUGACCUACGGCGUGCAGUGCUUCAGC<br>CGCUACCCCGACCACAUGAAGCAGCACGACUUCUUCAAGUCCGC<br>CAUGCCCGAAGGCUACGUCCAGGAGCGCACCAUCUUCUUCAAGG<br>ACGACGGCAACUACAAGACCCGCGCCGAGGUGAAGUUCGAGGGC<br>GACACCCUGGUGAACCGCAUCGAGCUGAAGGGCAUCGACUUCAA<br>GGAGGACGGCAACAUCCUGGGGCACAAGCUGGAGUACAACUACA<br>ACAGCCACAACGUCUAUAUCAUGGCCGACAAGCAGAAGAACGGC<br>AUCAAGGUGAACUUCAAGAUCCGCCACAACAUCGAGGACGGCAG

	CGUGCAGCUCGCCGACCACUACCAGCAGAACACCCCCAUCGGCGACGGCCCCGUGCUGCUGCCCGACAACCACUACCUGAGCACCCAGUCCGCCCUGAGCAAAGACCCCAACGAGAAGCGCGAUCACAUGGUCCUGCUGGAGUUCGUGACCGCCGCCGGGAUCACUCUCGGCAUGGACGAGCUGUACAAGUAA
luciferase	ATGGAAGACGCCAAAAACATAAAGAAAGGCCCGGCGCCATTCTATCCGCTGGAAGATGGAACCGCTGGAGAGCAACTGCATAAGGCTATGAAGAGATACGCCCTGGTTCCTGGAACAATTGCTTTTACAGATGCACATATCGAGGTGGACATCACTTACGCTGAGTACTTCGAAATGTCCGTTCGGTTGGCAGAAGCTATGAAACGATATGGGCTGAATACAAATCACAGAATCGTCGTATGCAGTGAAAACTCTCTTCAATTCTTTATGCCGGTGTTGGGCGCGTTATTTATCGGAGTTGCAGTTGCGCCCGCGAACGACATTTATAATGAACGTGAATTGCTCAACAGTATGGGCATTTCGCAGCCTACCGTGGTGTTCGTTTCCAAAAAGGGGTTGCAAAAAATTTTGAACGTGCAAAAAAAGCTCCCAATCATCCAAAAAATTATTATCATGGATTCTAAAACGGATTACCAGGGATTTCAGTCGATGTACACGTTCGTCACATCTCATCTACCTCCCGGTTTTAATGAATACGATTTTGTGCCAGAGTCCTTCGATAGGGACAAGACAATTGCACTGATCATGAACTCCTCTGGATCTACTGGTCTGCCTAAAGGTGTCGCTCTGCCTCATAGAACTGCCTGCGTGAGATTCTCGCATGCCAGAGATCCTATTTTTGGCAATCAAATCATTCCGGATACTGCGATTTTAAGTGTTGTTCCATTCCATCACGGTTTTGGAATGTTTACTACACTCGGATATTTGATATGTGGATTTCGAGTCGTCTTAATGTATAGATTTGAAGAAGAGCTGTTTCTGAGGAGCCTTCAGGATTACAAGATTCAAAGTGCGCTGCTGGTGCCAACCCTATTCTCCTTCTTCGCCAAAAGCACTCTGATTGACAAATACGATTTATCTAATTTACACGAAATTGCTTCTGGTGGCGCTCCCCTCTCTAAGGAAGTCGGGGAAGCGGTTGCCAAGAGGTTCCATCTGCCAGGTATCAGGCAAGGATATGGGCTCACTGAGACTACATCAGCTATTCTGATTACACCCGAGGGGGATGATAAACCGGGCGCGGTCGGTAAAGTTGTTCCATTTTTTGAAGCGAAGGTTGTGGATCTGGATACCGGGAAAACGCTGGGCGTTAATCAAAGAGGCGAACTGTGTGTGAGAGGTCCTATGATTATGTCCGGTTATGTAAACAATCCGGAAGCGACCAACGCCTTGATTGACAAGGATGGATGGCTACATTCTGGAGACATAGCTTACTGGGACGAAGACGAACACTTCTTCATCGTTGACCGCCTGAAGTCTCTGATTAAGTACAAAGGCTATCAGGTGGCTCCCGCTGAATTGGAATCCATCTTGCTCCAACACCCCAACATCTTCGACGCAGGTGTCGCAGGTCTTCCCGACGATGACGCCGGTGAACTTCCCGCCGCCGTTGTTGTTTTGGAGCACGGAAAGACGATGACGGAAAAAGAGATCGTGGATTACGTCGCCAGTCAAGTAACAACCGC

	GAAAAAGTTGCGCGGAGGAGTTGTGTTTGTGGACGAAGTACCGAA<br>AGGTCTTACCGGAAAACTCGACGCAAGAAAAATCAGAGAGATCCT<br>CATAAAGGCCAAGAAGGGCGGAAAGATCGCCGTGTAA
